# Biopsychosocial Determinants and Comorbid Risks of Obesity Among University Students: A Cross-Sectional Study

**DOI:** 10.3390/healthcare13141736

**Published:** 2025-07-18

**Authors:** Osama Albasheer, Mohamed Salih Mahfouz, Turki I. Aljezani, Mohammed Hassan Ghasham, Idris Harun Samily, Majid Muhammad Hakami, Naif Muslih Alshamrani, Shaima Abdu Hantul, Haneen A. Almutairi, Amal H. Mohamed, Nagla Abdalghani, Lamyaa A. M. El Hassan, Gassem Gohal, Ali Ali Ahmad Al-Makramani, Abdelkhalig Elhilu

**Affiliations:** 1 Family Medicine, Family and Community Medicine Department, Faculty of Medicine, Jazan University, Jazan 45142, Saudi Arabia; drosama802@gmail.com; 2 Faculty of Medicine, Jazan University, Jazan 45142, Saudi Arabia; turk3y90@gmail.com (T.I.A.); endreas14@gmail.com (I.H.S.); 202200989@stu.jazanu.edu.sa (M.M.H.);; 3 Internal Medicine, East Jeddah General Hospital, Jeddah 22253, Saudi Arabia; 4 Internal Medicine Department, Faculty of Medicine, Jazan University, Jazan 45142, Saudi Arabia; amalhm2010@hotmail.com; 5 Respiratory Therapy Department, Faculty of Nursing and Health Sciences, Jazan University, Jazan 45142, Saudi Arabia; nabdalghani@jazanu.edu.sa; 6 Department of Basic Medical Sciences, Faculty of Medicine, Jazan University, Jazan 45142, Saudi Arabia; lelhassan@jazanu.edu.sa; 7 Department of Pediatric Medicine, Faculty of Medicine, Jazan University, Jazan 45142, Saudi Arabia; ggohal@jazanu.edu.sa (G.G.); makra3@yahoo.com (A.A.A.A.-M.); 8 Department of Surgery, Faculty of Medicine, Jazan University, Jazan 45142, Saudi Arabia

**Keywords:** university students, biopsychosocial factors, comorbidities, Saudi Arabia

## Abstract

**Background/Objectives:** Obesity among university students is a growing concern, often influenced by biological, psychological, and social factors. Few studies in Saudi Arabia have addressed this issue using a comprehensive framework. This study aims to examine the prevalence of obesity and its biopsychosocial predictors among university students, as well as their perceptions, behaviors, and comorbidities. **Methods:** A cross-sectional study was conducted at Jazan University during the 2024–2025 academic year. A total of 819 undergraduate students completed a structured, self-administered Arabic questionnaire. The tool assessed sociodemographic variables, body mass index (BMI) (calculated from self-reported height and weight), biological and psychological factors, social influences, lifestyle behaviors, and comorbidities. Bivariate associations were tested using chi-square analyses, and multivariate logistic regression was used to identify independent predictors of obesity. **Results:** The prevalence of obesity was 19.6%, and 22.6% of students were overweight. Obesity was significantly more prevalent among males (26.7%) than females (9.6%, *p* < 0.001) and among students aged 24 years and above (24.0%, *p* = 0.024). Independent predictors of obesity included being overweight in childhood (AOR = 5.23, 95% CI: 3.47–7.90), belief in a genetic predisposition (AOR = 4.66), emotional eating (AOR = 2.57), academic or personal stress (AOR = 5.36), and social pressures related to body image (AOR = 2.96). Comorbidities significantly associated with obesity included high cholesterol (AOR = 5.40), sleep disorders (AOR = 2.99), and joint pain (AOR = 1.96). More than 80% of students with obesity reported current or past weight loss attempts, and nearly 60% received medical advice to lose weight. **Conclusions:** Obesity among Jazan University students is significantly associated with male gender, early-life weight history, emotional and academic stress, and social pressures. Students with obesity also experience a higher burden of comorbid conditions, even at a young age. These findings highlight the need for integrated, student-centered interventions that address both the psychological and social dimensions of weight management in university settings.

## 1. Introduction

Obesity has rapidly emerged as one of the foremost public health challenges of the 21st century, owing to its complex etiology and far-reaching impact on physical and mental well-being [1]. The condition is no longer viewed solely as an outcome of excessive caloric intake but rather as a multifactorial disorder influenced by a combination of biological, psychological, and social determinants [2,3,4]. In this context, the biopsychosocial model, as originally proposed by Engel, offers a holistic framework that is particularly useful for understanding the intricate factors contributing to obesity [5]. It integrates biological factors (e.g., genetics, early-life weight history), psychological influences (e.g., stress, emotional eating), and social determinants (e.g., peer pressure, body image norms) [6]. Applying this model allows researchers to move beyond reductionist approaches and consider the interplay of internal and external influences on obesity-related behaviors.

University students represent a unique and vulnerable population in which the risk of obesity is often heightened by significant lifestyle changes [7]. Transitioning to university life typically involves increased academic demands, altered eating patterns, and reduced physical activity, all of which may contribute to weight gain [8,9]. Furthermore, the psychological stresses associated with this period, such as anxiety, depression, and body image concerns, can exacerbate unhealthy eating behaviors and further complicate weight management efforts [10].

Although prior research has explored psychological and dietary correlates of obesity among university students, such as a study by El Ansari et al. (2014) [11], who examined food patterns and stress in a UK–Egypt student sample, and a study by Suwalska et al. (2022) [12], who assessed eating behaviors and depressive symptoms in Polish healthcare students, few studies have integrated biological, psychological, and social variables within a single biopsychosocial framework in the Middle Eastern context. To our knowledge, this study is among the first in Saudi Arabia to do so, focusing specifically on self-reported biopsychosocial factors alongside obesity prevalence and comorbidities in a university population.

Understanding how students perceive their own weight, recognize the risk factors, and acknowledge the potential complications, such as diabetes, cardiovascular diseases, and metabolic syndrome, could provide critical insights into why obesity persists in this important segment of the population. Such insights are essential for designing targeted and effective interventions to improve health outcomes in university settings.

## 2. Materials and Methods

### 2.1. Study Design and Setting

This study employed a cross-sectional design to explore the biopsychosocial determinants of obesity, personal perceptions, and associated comorbidities among university students during the 2024–2025 academic year. The research was conducted at Jazan University, a major academic institution in the southern region of Saudi Arabia, which was founded in 2006. According to the annual report published by Jazan University, the total student population is approximately 45,000.

### 2.2. Study Population

The target population included undergraduate students at Jazan University from selected faculties, including health-related, scientific, and humanities disciplines. Eligible participants were current undergraduate students aged 18 years or older who consented to voluntarily complete the questionnaire. There were no specific exclusion criteria other than failure to provide informed consent.

### 2.3. Sample Size and Sampling

Using the Raosoft sample size calculator (http://www.raosoft.com/samplesize.html, accessed on 20 December 20024) and assuming a total population of 45,000 students, a 95% confidence level, 50% response distribution, and a margin of error of 4%, the minimum required sample size was estimated at approximately 593 students. To enhance statistical power and accommodate potential subgroup analyses, as well as to account for an anticipated 20% non-response rate, the target sample size was increased to approximately 711 students. During data collection, 819 completed responses were collected. Participants were recruited using a non-probability convenience sampling method through faculty WhatsApp groups and student networks.

### 2.4. Tool and Recruitment

Data were collected using a self-administered online questionnaire developed in Google Forms. The instrument was developed in Arabic and reviewed by subject matter experts to ensure content validity, clarity, and cultural appropriateness. The questionnaire covered several key domains, including sociodemographic information (such as age, gender, academic level, and college affiliation), self-reported height and weight for body mass index (BMI) calculation, and a series of items assessing biological, psychological, and social factors contributing to obesity. Additional sections addressed students’ perceptions and knowledge of obesity-related health risks, lifestyle behaviors, weight loss attempts, and any medical interventions received (a copy of the study questionnaire can be found in the Supplementary Materials section).

To evaluate the feasibility and reliability of the questionnaire, a pilot study was conducted with 30 medical students. These participants were excluded from the final analysis. The pilot testing process confirmed the clarity and consistency of the tool, yielding a Cronbach’s alpha of 0.78, indicating acceptable internal reliability. Minor modifications were made based on pilot feedback to enhance the questionnaire’s comprehensibility and improve overall response rates.

Participants were recruited using a combination of campus-based outreach efforts and digital platforms targeting university students (e.g., WhatsApp, Telegram, X). Prior to participation, all students were provided with detailed information about the study and gave informed consent. To promote candidness and minimize social desirability bias, responses were collected anonymously.

### 2.5. Data Analysis and Interpretation

Data were coded, cleaned, and analyzed using IBM SPSS Statistics version 29. Descriptive statistics were used to summarize the participants’ demographic characteristics, BMI categories, and responses to the questionnaire items. Body mass index (BMI) was calculated using self-reported height and weight, applying the standard formula (weight in kilograms divided by height in meters squared). Participants were then categorized into four weight groups according to the classification established by the World Health Organization (WHO) [13]. These categories included underweight (BMI less than 18.5), normal weight (BMI between 18.5 and 24.9), overweight (BMI between 25.0 and 29.9), and obese (BMI of 30.0 or higher). These classifications formed the basis for analyzing the distribution and patterns of obesity among students.

Associations between BMI categories and various sociodemographic, behavioral, and psychosocial variables were examined using chi-square tests. Variables that demonstrated statistically significant associations with obesity in the bivariate analyses were entered into a multivariate logistic regression model to identify independent predictors of obesity. The model yielded adjusted odds ratios (AORs) with 95% confidence intervals to quantify the strength and direction of associations. Statistical significance was determined at a *p*-value of less than 0.05. To ensure the reliability of the model estimates, multicollinearity was assessed using the variance inflation factor (VIF), confirming that no problematic intercorrelations existed among predictor variables.

### 2.6. Ethical Considerations

Ethical approval for this study was obtained from the Standing Committee for Scientific Research at Jazan University, Kingdom of Saudi Arabia (IRB Registration No. HAPO-10-Z-001; Reference No. REC-45/05/861, dated 4 December 2023). Additional authorization for data collection was granted by the Faculty of Medicine. The study was conducted in accordance with recognized ethical guidelines applicable to the jurisdiction of Saudi Arabia. Informed consent was obtained from all participants prior to data collection. Participation was voluntary, and strict measures were implemented to ensure anonymity and confidentiality throughout the study. No identifiable personal information was collected or stored.

## 3. Results

### 3.1. Participant Characteristics and Obesity Patterns

Table 1 presents the background characteristics of the study population (*n* = 819) and the distribution of BMI categories across various demographic and health-related factors. As shown in Table 1, the overall prevalence of obesity among participants was 19.6%, highlighting a significant public health concern within this university-aged population. Table 1 further illustrates the BMI distribution: 44.8% of students had normal weight, 22.6% were overweight, and 13.0% were underweight.

From Table 1, the majority of participants were aged between 21–23 years (48.8%), followed by 18–20 years (39.4%), and those aged 24 and above (11.7%). Obesity was more prevalent among older age groups, particularly those aged ≥24 years, where 24.0% were obese compared to 16.7% in the youngest group (*p* = 0.024). Gender was significantly associated with obesity, as 26.7% of males were obese compared to only 9.6% of females (*p* < 0.001). Academic level was also related to obesity prevalence, with fifth-year students showing the highest obesity rate (32.6%) followed by third-year students (19.4%) (*p* = 0.017). Students from science colleges had a notably higher obesity rate (31.3%) compared to their peers in health-related (15.8%) and humanities and arts colleges (18.4%) (*p* = 0.001). Comorbidities such as high cholesterol (54.1% obese), sleep apnea (39.7% obese), and joint pain (27.8% obese) were significantly associated with obesity (*p* < 0.001, *p* = 0.001, and *p* = 0.003; respectively). Although a trend was observed for high blood pressure, the association was marginal (*p* = 0.051). Diabetes was not significantly related to obesity (*p* = 0.949).

### 3.2. Association Between Biopsychosocial Factors and Obesity

Table 2 presents the associations between self-reported biological, psychological, and social factors and obesity status among the participants. All the assessed personal biological factors—including beliefs about genetic predisposition, history of childhood overweight, and tendency to gain weight easily—were significantly associated with obesity (*p* < 0.001 for all). For instance, 45.2% of those who believed their obesity was due to genetics were obese, compared to only 15.1% of those who disagreed.

Among psychological factors, academic and personal stressors were strongly associated with obesity. Of those who agreed that stress influenced their weight, 88.0% were obese compared to 12.0% of those who disagreed (*p* < 0.001). Emotional eating (79.3% obese), psychological distress (83.6% obese), and body image concerns (74.1% obese) were also significantly linked to obesity (*p* < 0.001 for all). Regarding social factors, students who believed their social environment influenced their eating habits were more likely to be obese (76.7% vs. 23.3%, *p* = 0.010). Similarly, those who found it difficult to manage diet during social events (77.3% obese) and those affected by societal body image expectations (73.5% obese) reported significantly higher obesity rates (*p* = 0.019 and *p* < 0.001, respectively).

### 3.3. Perceptions, Lifestyle, and Weight Management Practices

Table 3 presents the relationship among students′ personal perceptions, lifestyle behaviors, weight loss attempts, and obesity. Although general awareness of obesity′s health effects was high among all students, certain perceptions were more pronounced in obese individuals. Notably, a greater proportion of obese students reported seeking information about obesity (83.5% vs. 69.1%, *p* = 0.002) and understanding effective weight management strategies (93.8% vs. 87.3%, *p* = 0.040).

In terms of lifestyle habits, there were no significant associations with physical activity, fast food intake, or sugary beverage consumption between obese and non-obese participants. However, obese students were significantly less likely to consume fruits and vegetables daily (47.0% vs. 60.0%, *p* = 0.010). Weight loss behaviors showed clear distinctions. A large proportion of obese students had tried to lose weight in the past year (85.3% vs. 53.1%, *p* < 0.001), were currently attempting weight loss (81.6% vs. 49.4%, *p* < 0.001), and expressed high motivation to lose weight (92.3% vs. 62.4%, *p* < 0.001). They were also more likely to have received medical advice to lose weight (58.9% vs. 18.4%, *p* < 0.001) or been prescribed weight-related treatment (23.3% vs. 9.8%, *p* < 0.001).

### 3.4. Multivariate Logistic Regression Analysis of Predictors of Obesity

Table 4 presents the multivariate logistic regression model identifying independent predictors of obesity. After adjusting for confounding variables, male gender remained a strong predictor of obesity, with males being over three times more likely to be obese than females (AOR = 3.42, 95% CI: 2.26–5.16, *p* < 0.001). Among comorbidities, high cholesterol had the strongest association (AOR = 5.40, 95% CI: 2.76–10.56, *p* < 0.001), followed by sleep apnea (AOR = 2.99, 95% CI: 1.71–5.23, *p* < 0.001) and joint pain (AOR = 1.96, 95% CI: 1.36–2.82, *p* < 0.001). High blood pressure also showed a significant association (AOR = 2.08, *p* = 0.030), while diabetes did not.

In the biological domain, students who were overweight since childhood (AOR = 5.23), believed in a genetic cause (AOR = 4.66), or gained weight easily (AOR = 3.76) were significantly more likely to be obese (*p* < 0.001 for all). Psychological factors such as emotional distress (AOR = 2.62), emotional eating (AOR = 2.57), and low self-esteem or body image concerns (AOR = 2.96) were also independently associated with obesity. Academic stress remained a strong predictor (AOR = 5.36, *p* < 0.001). From the social context, societal expectations (AOR = 2.61), peer attitudes (AOR = 2.96), and the social environment (AOR = 1.83) were all significant.

Motivation and behavior-related factors had some of the highest odds ratios. Students who were motivated to lose weight (AOR = 7.24), attempted weight loss in the past year (AOR = 5.12), received healthcare advice (AOR = 6.35), or were prescribed weight-related medications (AOR = 2.79) had significantly increased odds of being obese.

## 4. Discussion

This study aimed to examine the prevalence and predictors of obesity among university students through a biopsychosocial lens, while also evaluating students′ personal perceptions, lifestyle behaviors, and related comorbidities. The findings revealed that nearly one in five students were classified as obese, and over two in five had excess weight when combining both overweight and obese categories. These findings underscore the significance of obesity as an emerging public health issue among young adults in academic settings.

One of the key findings was the higher likelihood of obesity among male students, who were more than three times as likely to be obese as their female counterparts. This gender disparity aligns with findings from some regional studies that suggest male university students may be more prone to unhealthy dietary behaviors and less likely to perceive excess weight as a health issue [14]. In contrast, Althumiri et al. reported higher obesity rates among females in the general Saudi population, attributing the disparity to factors such as physical inactivity, sociocultural constraints, and biological predispositions [15]. However, among university students, especially those in health-related faculties, these patterns may differ. Male students may have greater access to high-calorie foods, lower engagement in preventive health behaviors, and may underreport concerns about body weight due to social or cultural norms. Additionally, age, academic stress, and lifestyle factors specific to this population may help explain the observed differences. Supporting this, Syam’s study at Majmaah University using the Student Lifestyle and Obesity Risk Questionnaire (SLORQ) found a high prevalence of obesity linked to poor dietary habits, limited physical activity, and inadequate sleep [16]. However, that study did not observe significant gender or age-related differences in BMI, underscoring the variability in obesity patterns across different university settings and emphasizing the need for further gender-specific investigations.

Older students in our study, particularly those in their final academic years, demonstrated a higher likelihood of obesity. This trend is consistent with findings from a Belgian study, which reported a progressive increase in overweight and obesity prevalence across academic levels among university students [17]. Similarly, longitudinal research from the United States showed that BMI steadily increased from freshman to senior year, supporting the notion that weight gain during university may be cumulative [18]. These patterns may reflect the compounding effects of academic stress, reduced physical activity, and unhealthy lifestyle adaptations over the course of university education.

Interestingly, students from science colleges had higher obesity rates than those from health-related or humanities programs. This may be attributed to discipline-specific lifestyle differences, such as increased study load, irregular schedules, or less exposure to health education [19]. Previous studies have shown that students in health-related fields often display better awareness and more positive attitudes toward nutrition and physical activity [20,21].

The study also found strong associations between obesity and early-life biological factors, such as a personal history of being overweight during childhood and having family members with obesity. These findings are consistent with global research showing that childhood overweight and familial tendencies substantially increase the risk of adult obesity [22,23,24]. In contrast, while many students perceived hormonal imbalances as a factor contributing to their weight, this belief was not a significant predictor in multivariate analysis, highlighting a possible misconception among students.

From a psychological standpoint, the role of emotional and academic stress emerged as a major contributor to obesity. Students who reported struggling with emotional eating, stress-related food behaviors, or low self-esteem were significantly more likely to be obese. Nearly nine out of ten obese students acknowledged that stress and emotions played a role in their weight gain. These findings align with the literature showing that psychological distress can play a mediating role in obesity through mechanisms such as emotional eating, hormonal imbalance, and disrupted circadian rhythms [2,25,26].

Social factors also played a critical role. Students with obesity were more likely to report that social gatherings, peer influence, and societal body image expectations impacted their ability to manage weight. The influence of cultural and social norms in shaping health behaviors among youth has been documented in both local and international studies [3,27], and the present findings reinforce the need for culturally sensitive, community-based interventions.

Although fast food and sugary beverage consumption were widely prevalent in our sample, they were not significantly associated with obesity status. This finding challenges common assumptions but may be explained by a relatively uniform pattern of consumption among students or potential underreporting due to social desirability bias. A recent cross-sectional study conducted in Kuwait among 411 university students similarly found no significant association between sugar-sweetened beverage (SSB) intake and BMI after adjusting for gender, age, and health awareness, despite over 40% of students reporting daily soda consumption [28]. In contrast, a 2024 study of female university students in the United Arab Emirates reported a significant association between high intake of sugary beverages—including soft drinks, milk-based drinks, and energy drinks—and increased rates of overweight and obesity, suggesting that beverage choices may play a more prominent role in specific demographic subgroups [29].

Importantly, the study identified a clear link between obesity and several comorbid conditions, even among this young population. Students with high cholesterol, sleep disturbances, or joint pain were substantially more likely to be obese. These findings echo those of earlier studies conducted in the Middle East and North Africa (MENA) region, which have shown that obesity-related complications can emerge early and silently among adolescents and young adults [30].

Although the majority of students demonstrated good knowledge about obesity and its complications, this awareness did not consistently translate into healthy behavior. This disconnect between knowledge and action has been widely reported in the public health literature and emphasizes the importance of behavioral and motivational strategies rather than education alone [31].

Finally, students with obesity were significantly more likely to have attempted weight loss, with over four out of five reporting recent or ongoing efforts. Moreover, many of them indicated that these efforts were initiated following advice from healthcare providers. This finding supports the critical role of medical counseling in triggering lifestyle change and aligns with existing evidence that physician engagement enhances patient motivation [32,33].

Strengths, Limitations, and Future Directions

This study provides a comprehensive and multidimensional perspective on obesity among university students by integrating biological, psychological, and social factors within a single analytical framework. Unlike studies that narrowly focus on lifestyle behaviors or demographic predictors, this research adopts a holistic approach rooted in the biopsychosocial model, allowing for a richer understanding of the complex interplay of influences on student health. The relatively large and diverse sample drawn from multiple colleges and academic levels further enhances the generalizability of the findings within the university context. In addition, this study identifies a clear and measurable association between obesity and multiple comorbid conditions, such as high cholesterol, sleep disturbances, and joint pain, even among a relatively young population. This finding is particularly significant because it illustrates that the health consequences of obesity are not limited to older adults but can emerge silently and progressively during the university years. By documenting these early complications in a cross-sectional academic setting, the study contributes valuable evidence to the growing recognition that adolescence and young adulthood are critical windows for the onset of chronic, obesity-related conditions.

However, the study is not without limitations. Given its cross-sectional design, it does not allow for causal inference, and associations observed between variables and obesity status should be interpreted accordingly. The reliance on self-reported height and weight may have introduced measurement bias, particularly underreporting of weight or overreporting of height, which could influence BMI classification. Moreover, while efforts were made to ensure representation across academic disciplines, the participants were recruited from selected faculties using convenience sampling. This may limit the generalizability of the findings. The use of a structured questionnaire also means that some nuances, particularly in students’ psychological experiences or social pressures, may not have been fully captured. Although gender differences in obesity prevalence were observed, our analysis did not explore gender-specific patterns in biopsychosocial determinants or comorbidities. Future research with stratified models or larger gender-balanced samples is warranted to investigate whether these factors differ significantly between males and females.

Looking ahead, future research would benefit from longitudinal designs that can track weight trajectories and behavioral changes over time, offering greater insight into the development and persistence of obesity during the university years. Expanding the scope to include multiple institutions or regions would improve generalizability and allow for comparative analysis across diverse educational and cultural settings. Additionally, integrating qualitative methods, such as focus groups or interviews, could deepen the understanding of students′ lived experiences and contextual influences that quantitative tools may overlook. There is also scope to explore the effectiveness of targeted interventions, particularly those that address mental health, social norms, and institutional support systems, in mitigating obesity risk among university populations.

## 5. Conclusions

This study underscores the multifactorial nature of obesity among university students, revealing that excess weight is not solely a product of individual lifestyle choices but is deeply rooted in biological predispositions, psychological stressors, and social influences. The use of a biopsychosocial framework revealed significant associations among obesity and early-life weight history, emotional and academic pressures, body image concerns, and socially driven eating behaviors. Moreover, comorbidities such as high cholesterol, joint pain, and sleep disorders were notably more common among obese students, indicating that the physical burden of obesity is already manifesting in this young population.

Despite a generally high level of knowledge regarding obesity and its health risks, many students continued to engage in behaviors that contribute to weight gain. This disconnect highlights the limitations of awareness campaigns alone and points to the need for more integrated, behaviorally informed interventions. The fact that a substantial proportion of obese students reported receiving medical advice or actively attempting to lose weight suggests that clinical encounters can serve as critical intervention points.

Based on these findings, several tailored recommendations can be made. Universities should implement multidisciplinary health promotion programs that combine nutritional education, psychological counseling, and peer support. These initiatives should particularly target students reporting high stress levels or body image dissatisfaction, as they are among the most vulnerable. Embedding weight and lifestyle screening into student health services can facilitate early identification and personalized support. Moreover, educational interventions should be adapted to address not only knowledge but also motivation, coping strategies, and social norms around eating and body image. Incorporating these strategies within university health systems will not only support individual students in managing their weight but also contribute to long-term public health gains by interrupting the trajectory of obesity and its associated complications early in adulthood.

## Figures and Tables

**Table 1:** Study participants′ background characteristics and obesity pattern among Jazan University students (*n* = 819).

Characteristics		All Participants		BMI Categories								*p* Value
				Underweight		Normal Weight		Overweight		Obese		
		N	%	N	%	N	%	N	%	N	%	
Age groups	18–20 years	323	(39.4)	62	(19.2)	143	(44.3)	64	(19.8)	54	(16.7)	0.024
	21–23 years	400	(48.8)	47	(11.8)	181	(45.3)	89	(22.3)	83	(20.8)	
	24 years and more	96	(11.7)	9	(9.4)	37	(38.5)	27	(28.1)	23	(24.0)	
Gender	Male	476	(58.1)	47	(9.9)	184	(38.7)	118	(24.8)	127	(26.7)	<0.001
	Female	343	(41.9)	71	(20.7)	177	(51.6)	62	(18.1)	33	(9.6)	
Academic level	1st year	152	(18.6)	27	(17.8)	70	(46.1)	29	(19.1)	26	(17.1)	0.017
	2nd year	111	(13.6)	26	(23.4)	46	(41.4)	18	(16.2)	21	(18.9)	
	3rd year	129	(15.8)	13	(10.1)	58	(45.0)	33	(25.6)	25	(19.4)	
	4th year	241	(29.4)	33	(13.7)	109	(45.2)	58	(24.1)	41	(17.0)	
	5th year	95	(11.6)	6	(6.3)	35	(36.8)	23	(24.2)	31	(32.6)	
	6th year	91	(11.1)	13	(14.3)	43	(47.3)	19	(20.9)	16	(17.6)	
Colleges	Health Related	500	(61.1)	70	(14.0)	234	(46.8)	117	(23.4)	79	(15.8)	0.001
	Science Colleges	192	(23.4)	28	(14.6)	62	(32.3)	42	(21.9)	60	(31.3)	
	Humanity and Arts	87	(10.6)	13	(14.9)	44	(50.6)	14	(16.1)	16	(18.4)	
	Not Stated	40	(4.9)	7	(17.5)	21	(52.5)	7	(17.5)	5	(12.5)	
Diagnosed with high blood pressure	Yes	43	(5.3)	3	(7.0)	14	(32.6)	12	(27.9)	14	(32.6)	0.051
	No	776	(94.7)	115	(14.8)	347	(44.7)	168	(21.6)	146	(18.8)	
Diagnosed with diabetes mellitus	Yes	35	(4.3)	5	(14.3)	14	(40.0)	8	(22.9)	8	(22.9)	0.949
	No	784	(95.7)	113	(14.4)	347	(44.3)	172	(21.9)	152	(19.4)	
Diagnosed with high cholesterol	Yes	37	(4.5)	3	(8.1)	8	(21.6)	6	(16.2)	20	(54.1)	<0.001
	No	782	(95.5)	115	(14.7)	353	(45.1)	174	(22.3)	140	(17.9)	
Diagnosed with sleep apnea or other sleep disorders	Yes	58	(7.1)	5	(8.6)	17	(29.3)	13	(22.4)	23	(39.7)	0.001
	No	761	(92.9)	113	(14.8)	344	(45.2)	167	(21.9)	137	(18.0)	
Experienced joint pain or discomfort that affects the daily activities	Yes	223	(27.2)	30	(13.5)	83	(37.2)	48	(21.5)	62	(27.8)	0.003
	No	596	(72.8)	88	(14.8)	278	(46.6)	132	(22.1)	98	(16.4)	
All participants		819	(100)	118	(14.4)	361	(44.1)	180	(22.0)	160	(19.5)	

**Table 2:** Association between biopsychosocial factors and obesity among Jazan University students (*n* = 819).

Statements		Response	Obesity				*p* Value
			No		Yes		
			N	%	N	%	
Personal Biological Factors	I believe I am overweight or obese due to genetic or family history factors.	Disagree	451	(84.9)	69	(54.8)	<0.001
		Agree	80	(15.1)	57	(45.2)	
	I have struggled with being overweight since childhood.	Disagree	502	(85.5)	70	(53.0)	<0.001
		Agree	85	(14.5)	62	(47.0)	
	I gain weight easily, even when I make an effort to control my diet and exercise.	Disagree	317	(61.9)	35	(30.2)	<0.001
		Agree	195	(38.1)	81	(69.8)	
	I believe hormonal imbalances (e.g., thyroid, insulin) are contributing to my weight gain.	Disagree	242	(48.6)	46	(40.0)	0.096
		Agree	256	(51.4)	69	(60.0)	
Personal Psychological Factors	I believe academic or personal pressures (e.g., workload, exams, family issues) have contributed to my weight gain or difficulty losing weight.	Disagree	225	(42.3)	16	(12.0)	<0.001
		Agree	307	(57.7)	117	(88.0)	
	I believe my weight gain or difficulty losing weight is related to emotional or psychological factors (e.g., stress, anxiety, depression).	Disagree	179	(34.0)	22	(16.4)	<0.001
		Agree	348	(66.0)	112	(83.6)	
	I tend to overeat or eat unhealthy foods when I am stressed, anxious, or feeling down.	Disagree	205	(40.1)	25	(20.7)	<0.001
		Agree	306	(59.9)	96	(79.3)	
	My emotions or mental state (e.g., low self-esteem, body image concerns) make it hard for me to focus on managing my weight.	Disagree	250	(50.8)	30	(25.9)	<0.001
		Agree	242	(49.2)	86	(74.1)	
Personal Social Factors	I believe my social environment (e.g., family, friends, or community) has influenced my eating habits or weight gain.	Disagree	178	(35.7)	28	(23.3)	0.010
		Agree	320	(64.3)	92	(76.7)	
	Social gatherings or events make it difficult for me to maintain a healthy diet or manage my weight.	Disagree	173	(33.4)	29	(22.7)	0.019
		Agree	345	(66.6)	99	(77.3)	
	I feel that societal expectations or pressures about body image have affected how I view my own weight.	Disagree	240	(48.6)	30	(26.5)	<0.001
		Agree	254	(51.4)	83	(73.5)	
	I believe the attitudes of people around me (e.g., family, friends, coworkers) have impacted my weight or my efforts to lose weight.	Disagree	255	(53.5)	31	(27.9)	0.001

**Table 3:** Association between personal perception, lifestyle, and weight loss attempts and obesity among Jazan University students (*n* = 819).

Statements		Response	Obesity				*p* Value
			No		Yes		
			N	%	N	%	
Personal Perception and Knowledge of My Own Obesity	I understand what obesity is and how it affects my overall health.	Disagree	40	(6.8)	5	(3.5)	0.143
		Agree	551	(93.2)	138	(96.5)	
	I am aware of the risk factors (e.g., poor diet, lack of exercise, genetics) that can lead to obesity.	Disagree	43	(7.4)	9	(6.2)	0.628
		Agree	541	(92.6)	136	(93.8)	
	I know about the potential complications of obesity, such as diabetes, heart disease, or joint problems.	Disagree	38	(6.5)	5	(3.6)	0.192
		Agree	550	(93.5)	135	(96.4)	
	I believe I can take steps to reduce the health risks associated with my weight.	Disagree	49	(8.8)	5	(3.8)	0.060
		Agree	509	(91.2)	125	(96.2)	
	I have a clear understanding of the best practices for managing my weight (e.g., diet, exercise, lifestyle changes).	Disagree	69	(12.7)	8	(6.3)	0.040 *
		Agree	475	(87.3)	120	(93.8)	
	I feel confident in my ability to make informed decisions about my health and weight.	Disagree	70	(13.3)	19	(15.3)	0.557
		Agree	456	(86.7)	105	(84.7)	
	I regularly seek out information about obesity, its causes, and how to manage it effectively.	Disagree	150	(30.9)	20	(16.5)	0.002 *
		Agree	336	(69.1)	101	(83.5)	
Lifestyle and Habits	I engage in regular physical activity (e.g., exercise, sports) at least 3 times a week.	Disagree	249	(49.5)	68	(53.5)	0.416
		Agree	254	(50.5)	59	(46.5)	
	I often consume fast food or processed foods as part of my diet.	Disagree	123	(24.3)	30	(24.2)	0.979
		Agree	383	(75.7)	94	(75.8)	
	I pay attention to portion sizes when I eat.	Disagree	242	(50.2)	60	(55.6)	0.315
		Agree	240	(49.8)	48	(44.4)	
	I try to include fruits and vegetables in my daily meals.	Disagree	197	(40.0)	62	(53.0)	0.010 *
		Agree	296	(60.0)	55	(47.0)	
	I typically drink sugary beverages (e.g., soda, energy drinks) regularly.	Disagree	251	(49.7)	54	(43.9)	0.248
		Agree	254	(50.3)	69	(56.1)	
Weight Loss Attempts	I have tried to lose weight in the past year.	Disagree	254	(46.9)	20	(14.7)	<0.001
		Agree	288	(53.1)	116	(85.3)	
	I am currently trying to lose weight.	Disagree	273	(50.6)	25	(18.4)	<0.001
		Agree	266	(49.4)	111	(81.6)	
	I feel motivated to lose weight for health or appearance reasons.	Disagree	207	(37.6)	11	(7.7)	<0.001
		Agree	343	(62.4)	132	(92.3)	
Medical Interventions and Advice	A healthcare provider has advised me to lose weight for health reasons.	Disagree	465	(81.6)	53	(41.1)	<0.001
		Agree	105	(18.4)	76	(58.9)	
	I have been prescribed medication or treatment to address health issues related to my weight.	Disagree	524	(90.2)	102	(76.7)	<0.001
		Agree	57	(9.8)	31	(23.3)	

* Mean significant values.

**Table 4:** Logistic regression analysis of the factors associated with obesity among Jazan University students (*n* = 819).

Category	Factor	B	SE	Wald	*p* Value	COR	95 CI for COR	
							Lower Bound	Upper Bound
Gender	Male	1.229	0.210	34.130	0.000	3.418	2.263	5.163
Age Groups	18–20 years (ref)					1		
	21–23 years	0.266	0.193	1.885	0.170	1.304	0.893	1.906
	24 years and more	0.451	0.282	2.559	0.110	1.570	0.903	2.727
Comorbidity	high blood pressure (Yes)	0.734	0.338	4.710	0.030	2.083	1.074	4.042
	Having Diabetes (Yes)	0.209	0.413	0.256	0.613	1.232	0.549	2.765
	Diagnosed with high cholesterol levels (Yes)	1.685	0.343	24.172	<0.001	5.395	2.755	10.563
	Diagnosed with sleep apnea or other sleep disorders (Yes)	1.096	0.285	14.847	<0.001	2.993	1.714	5.228
	Experience joint pain or discomfort that affects the daily activities (Yes)	0.671	0.186	13.045	<0.001	1.957	1.359	2.817
Personal Biological Factors	I believe I am overweight or obese due to genetic or family history factors.	1.538	0.216	50.619	<0.001	4.657	3.048	7.115
	I have struggled with being overweight since childhood.	1.655	0.210	61.978	<0.001	5.231	3.465	7.897
	I gain weight easily, even when I make an effort to control my diet and exercise.	1.325	0.222	35.684	<0.001	3.762	2.436	5.811
	I believe academic or personal pressures have contributed to my weight gain or difficulty losing weight.	1.679	0.281	35.791	<0.001	5.359	3.092	9.289
Social and Psychological Factors	I believe my weight gain or difficulty losing weight is related to emotional or psychological factors.	0.963	0.251	14.746	<0.001	2.619	1.602	4.280
	I tend to overeat or eat unhealthy foods when I am stressed, anxious, or feeling down.	0.945	0.242	15.246	<0.001	2.573	1.601	4.134
	My emotions or mental state (e.g., low self-esteem, body image concerns) make it hard for me to focus on managing my weight.	1.086	0.230	22.200	<0.001	2.961	1.885	4.652
	I believe my social environment (e.g., family, friends, or community) has influenced my eating habits or weight gain.	0.603	0.235	6.573	<0.001	1.828	1.153	2.898
	Social gatherings or events make it difficult for me to maintain a healthy diet or manage my weight.	0.538	0.231	5.426	0.020	1.712	1.089	2.691
	I feel that societal expectations or pressures about body image have affected how I view my own weight.	0.961	0.231	17.265	<0.001	2.614	1.661	4.113
	I believe the attitudes of people around me (e.g., family, friends, coworkers) have impacted my weight or my efforts to lose weight.	1.087	0.231	22.201	<0.001	2.964	1.886	4.658
Self-Desire to Lose Weight	I have tried to lose weight in the past year.	1.632	0.257	40.348	<0.001	5.115	3.091	8.464
	I feel motivated to lose weight for health or appearance reasons.	1.980	0.326	36.900	<0.001	7.242	3.823	13.718
	A healthcare provider has advised me to lose weight for health reasons.	1.849	0.209	78.192	<0.001	6.350	4.216	9.566
	I have been prescribed medication or treatment to address health issues related to my weight.	1.027	0.248	17.161	<0.001	2.794	1.718	4.543

Note: VIF = variance inflation factor; B = unstandardized regression coefficient; CI = confidence interval.

## Data Availability

The datasets used and/or analyzed during the current study are available from the corresponding author (OA) upon reasonable request.

## Supplementary Materials

The following supporting information can be downloaded at: https://www.mdpi.com/article/10.3390/healthcare13141736/s1. Questionnaire on Biopsychosocial Factors and Obesity among University Students.

## Abbreviations

The following abbreviations are used in this manuscript:

BMI	Body Mass Index
WHO	World Health Organization
CI	Confidence Interval
VIF	Variance Inflation Factor
