# Supplementary material for: Biopsychosocial Determinants and Comorbid Risks of Obesity Among University Students: A Cross-Sectional Study
Source: Healthcare (Basel). 2025 Jul 18;13(14):1736. doi: 10.3390/healthcare13141736 (PMC12294840; doi:10.3390/healthcare13141736)
Supplement: Supplementary file 1 [file healthcare-13-01736-s001.zip › healthcare-3692219-supplementary.pdf]

Questionnaire on Biopsychosocial Factors and Obesity among University Students

Section 1: Personal Biological Factors

1. **I believe I am overweight or obese due to genetic or family history factors.**
    - **Strongly Disagree**
    - **Disagree**
    - **Neutral**
    - **Agree**
    - **Strongly Agree**
2. **I have struggled with being overweight since childhood.**
    - **Strongly Disagree**
    - **Disagree**
    - **Neutral**
    - **Agree**
    - **Strongly Agree**
3. **I gain weight easily, even when I make an effort to control my diet and exercise.**
    - **Strongly Disagree**
    - **Disagree**
    - **Neutral**
    - **Agree**
    - **Strongly Agree**
4. **I believe hormonal imbalances (e.g., thyroid, insulin) are contributing to my weight gain.**
    - **Strongly Disagree**
    - **Disagree**
    - **Neutral**
    - **Agree**
    - **Strongly Agree**

Section 2: Personal Psychological Factors

1. **I believe academic or personal pressures (e.g., workload, exams, family issues) have contributed to my weight gain or difficulty losing weight.**
    - **Strongly Disagree**

- **Disagree**
- **Neutral**
- **Agree**
- **Strongly Agree**

2. **I believe my weight gain or difficulty losing weight is related to emotional or psychological factors (e.g., stress, anxiety, depression).**
   - **Strongly Disagree**
   - **Disagree**
   - **Neutral**
   - **Agree**
   - **Strongly Agree**
3. **I tend to overeat or eat unhealthy foods when I am stressed, anxious, or feeling down.**
   - **Strongly Disagree**
   - **Disagree**
   - **Neutral**
   - **Agree**
   - **Strongly Agree**
4. **My emotions or mental state (e.g., low self-esteem, body image concerns) make it hard for me to focus on managing my weight.**
   - **Strongly Disagree**
   - **Disagree**
   - **Neutral**
   - **Agree**
   - **Strongly Agree**

Section 3: Personal Social Factors

1. **I believe my social environment (e.g., family, friends, or community) has influenced my eating habits or weight gain.**
   - **Strongly Disagree**
   - **Disagree**
   - **Neutral**

- **Agree**
- **Strongly Agree**

2. **Social gatherings or events make it difficult for me to maintain a healthy diet or manage my weight.**
    - **Strongly Disagree**
    - **Disagree**
    - **Neutral**
    - **Agree**
    - **Strongly Agree**
3. **I feel that societal expectations or pressures about body image have affected how I view my own weight.**
    - **Strongly Disagree**
    - **Disagree**
    - **Neutral**
    - **Agree**
    - **Strongly Agree**
4. **I believe the attitudes of people around me (e.g., family, friends, coworkers) have impacted my weight or my efforts to lose weight.**
    - **Strongly Disagree**
    - **Disagree**
    - **Neutral**
    - **Agree**
    - **Strongly Agree**

Section 4: Personal Perception and Knowledge of My Own Obesity

1. **I understand what obesity is and how it affects my overall health.**
    - **Strongly Disagree**
    - **Disagree**
    - **Neutral**
    - **Agree**
    - **Strongly Agree**

2. **I am aware of the risk factors (e.g., poor diet, lack of exercise, genetics) that can lead to obesity.**
    - **Strongly Disagree**
    - **Disagree**
    - **Neutral**
    - **Agree**
    - **Strongly Agree**
3. **I know about the potential complications of obesity, such as diabetes, heart disease, or joint problems.**
    - **Strongly Disagree**
    - **Disagree**
    - **Neutral**
    - **Agree**
    - **Strongly Agree**
4. **I believe I can take steps to reduce the health risks associated with my weight.**
    - **Strongly Disagree**
    - **Disagree**
    - **Neutral**
    - **Agree**
    - **Strongly Agree**
5. **I have a clear understanding of the best practices for managing my weight (e.g., diet, exercise, lifestyle changes).**
    - **Strongly Disagree**
    - **Disagree**
    - **Neutral**
    - **Agree**
    - **Strongly Agree**
6. **I feel confident in my ability to make informed decisions about my health and weight.**
    - **Strongly Disagree**
    - **Disagree**
    - **Neutral**

- Agree
- Strongly Agree

7. **I regularly seek out information about obesity, its causes, and how to manage it effectively.**
   - **Strongly Disagree**
   - **Disagree**
   - **Neutral**
   - **Agree**
   - **Strongly Agree**

Section 5: Lifestyle and Habits

1. **I engage in regular physical activity (e.g., exercise, sports) at least 3 times a week.**
   - **Strongly Disagree**
   - **Disagree**
   - **Neutral**
   - **Agree**
   - **Strongly Agree**
2. **I often consume fast food or processed foods as part of my diet.**
   - **Strongly Disagree**
   - **Disagree**
   - **Neutral**
   - **Agree**
   - **Strongly Agree**
3. **I pay attention to portion sizes when I eat.**
   - **Strongly Disagree**
   - **Disagree**
   - **Neutral**
   - **Agree**
   - **Strongly Agree**
4. **I try to include fruits and vegetables in my daily meals.**
   - **Strongly Disagree**

- **Disagree**
- **Neutral**
- **Agree**
- **Strongly Agree**

5. **I typically drink sugary beverages (e.g., soda, energy drinks) regularly.**
   - **Strongly Disagree**
   - **Disagree**
   - **Neutral**
   - **Agree**
   - **Strongly Agree**

Section 6: Comorbidity Assessment (Self-Reported)

1. **I have been diagnosed with high blood pressure.**
   - **Yes**
   - **No**
2. **I have been diagnosed with diabetes.**
   - **Yes**
   - **No**
3. **I have been diagnosed with high cholesterol levels.**
   - **Yes**
   - **No**
4. **I have been diagnosed with sleep apnea or other sleep disorders.**
   - **Yes**
   - **No**
5. **I experience joint pain or discomfort that affects my daily activities.**
   - **Yes**
   - **No**

Section 7: Weight Loss Attempts

1. **I have tried to lose weight in the past year.**
   - **Strongly Disagree**

- **Disagree**
- **Neutral**
- **Agree**
- **Strongly Agree**

2. **I am currently trying to lose weight.**
    - **Strongly Disagree**
    - **Disagree**
    - **Neutral**
    - **Agree**
    - **Strongly Agree**
3. **I feel motivated to lose weight for health or appearance reasons.**
    - **Strongly Disagree**
    - **Disagree**
    - **Neutral**
    - **Agree**
    - **Strongly Agree**

Section 8: Medical Interventions and Advice

1. **A healthcare provider has advised me to lose weight for health reasons.**
    - **Strongly Disagree**
    - **Disagree**
    - **Neutral**
    - **Agree**
    - **Strongly Agree**
2. **I have been prescribed medication or treatment to address health issues related to my weight.**
    - **Strongly Disagree**
    - **Disagree**
    - **Neutral**
    - **Agree**
    - **Strongly Agree**

Demographic Information

- Age: __________
- Gender: __________
- Year of Study: __________
- Field of Study: __________
- Height: __________ (cm)
- Weight: __________ (kg)
